# Prevalence and factors associated with low back pain among health care workers in southwestern Saudi Arabia

**DOI:** 10.1186/s12891-019-2431-5

**Published:** 2019-02-08

**Authors:** Ibrahim Alnaami, Nabil J. Awadalla, Mona Alkhairy, Suleiman Alburidy, Abdulaziz Alqarni, Almohannad Algarni, Rawan Alshehri, Bodoor Amrah, Mishal Alasmari, Ahmed A. Mahfouz

**Affiliations:** 1 Department of Surgery, King Khalid University, Abha, Saudi Arabia; 2 Department of Family and Community Medicine, College of Medicine, King Khalid University, Abha, Saudi Arabia; 3 Community Medicine Department, College of Medicine, Mansoura University, Mansoura, Egypt; 4 Medical Intern, College of Medicine, King Khalid University, Abha, Saudi Arabia; 5 Department of Epidemiology, High Institute of Public Health, Alexandria University, Alexandria, Egypt

**Keywords:** Low Back pain, Health care workers, Saudi Arabia

## Abstract

**Background:**

The purpose was to measure the prevalence and related risk factors of low back pain (LBP) among health care workers (HCWs) at different levels of health care in southwestern Saudi Arabia.

**Methods:**

A cross-sectional study using a self-administered questionnaire was conducted among HCWs providing primary, secondary and tertiary health care services in the Aseer region, southwestern Saudi Arabia. The questionnaire collected data regarding having LBP in the past 12 months, socio-demographics, work conditions and history of chronic diseases, regular physical exercise and overexertional back trauma. Univariate and multivariable logistic regression analyses were performed.

**Results:**

Out of 740 participants, the overall prevalence of LBP in the past 12 months amounted to73.9% (95% CI: 70.7–77.0). The prevalence of LBP with neurological symptoms reached 50.0%. The prevalence of LBP necessitating medications and or physiotherapy was 40.5%, while the prevalence of LBP requiring medical consultation was 20%. Using multivariable logistic regression, the following risk factors were identified: working in secondary and tertiary hospitals (aOR = 1.32, 95% CI:1.01–1.76), increased BMI (aOR = 1.10, 95% CI:1.01–3.65), and positive history of overexertional back trauma (aOR = 11.52, 95% CI:4.14–32.08). On the other hand, practising regular physical exercise was a significant protective factor (aOR = 0.61, 95% CI: 0.42–0.89).

**Conclusions:**

LBP is a common problem among HCWs. Many preventable risk factors have been identified, including exertional back trauma, increased BMI and lack of regular physical exercise. Occupational health and safety programmes to build ergonomically safe working conditions and encourage regular physical exercise are needed.

**Electronic supplementary material:**

The online version of this article (10.1186/s12891-019-2431-5) contains supplementary material, which is available to authorized users.

## Background

Low back pain (LBP) is one of the most common complaints necessitating health care. It is the most frequent type of musculoskeletal disorders. Approximately more than half of the general population will search for care for LBP at some point in their lives. Worldwide, the prevalence of LBP among the general population ranges between 15 and 45% [1]. In Saudi Arabia, the prevalence of LBP among the general population is reported to be 18.8% [2].

Low back pain (LBP) is an important public health issue, being of widespread and of a considerable negative social, psychological, and economic influence. Frequently, it is more common among individuals with exhausting occupations; in the world, 37% of LBP is related to occupations in which professionals are exposed to vibrations or prolonged periods of standing, such as miners, health care workers (HCWs), and professional drivers. A greater proportion of LBP is concomitant with the repetitive or prolonged awkward postures, which professionals within these jobs often undertake [3].

LBP is considered one of the most important causes of morbidity among health care workers (HCWs) that affects their work, and 18.7% of them with chronic LBP were using analgesic and or pain-relief drugs [4]. Additionally, a study in Sweden among HCWs showed a higher prevalence of LBP amounting to 77% compared to many other occupational groups [5]. Similarly, in Taiwan, a study showed that 72% of HCWs had LBP [6]. This problem is associated with major personal and occupationally related consequences, including disability and frequent absenteeism. LBP might result in activity limitation and sick leave for greater than 50% of HCWs [7].

HCWs at different health care levels carry out a variety of workplace activities that expose them to a variety of factors associated with a greater probability of LBP development [8].A variety of workplace and personal factors have been implicated to increase the risk of LBP among HCWs [1].The reported personal risk factors included age, sex, smoking, obesity and poor health status. On the other hand, the reported workplace factors comprised increased muscular sprains and strains due to intense work activity; prolonged standing, sitting, lifting of heavy objects; and psychosocial stress [9–12].

In Saudi Arabia, the problem of LBP is not distinctive from that in other parts of the globe. A few reports have addressed the prevalence of LBP among HCWs in Saudi Arabia [13]. However, the assessments were incomplete in terms of details of the pain and associated factors. The aim of the current study was to measure the prevalence of LBP among all groups of HCWs in different health care levels in southwestern Saudi Arabia and to explore related risk factors.

## Methods

The present study was a descriptive, cross-sectional study using a self-administered questionnaire. The study targeted hospitals and primary health care centres in the Aseer region, southwestern Saudi Arabia. The study included physicians, dentists, nurses, paramedics, and other medical practitioners. Retired medical practitioners and those who were not practising clinical work were excluded from the study.

Using the WHO manual for sample size calculation in health sciences [14], with an estimate of 43% of LBP [15], absolute required precision of 4 and 95% confidence interval, the calculated minimal sample size was 589 HCWs.

A list of all employees who met the inclusion criteria (currently practising HCWs) was obtained from the Aseer region Directorate of Health, Saudi Arabian Ministry of Health. The sample was selected using a one stage stratified cluster sampling technique. Practitioners were stratified according to their job, and then from each stratum, a sample was selected from the different groups using systematic random sampling depending on a practitioner’s names list with proportional allocation technique.

A modified self-administered questionnaire used by Homaid et al. [13] was applied for data collection in the present study (attached). It included personal and socio-demographic job-related data of HCWs and their experience with LBP in the past 12 months. LBP was defined as “pain, muscle tension, or stiffness localized below the costal margin and above the inferior gluteal folds, with or without leg pain (sciatica)” [16].The questionnaire also included data regarding work conditions (standing time, lifting, transferring, or pulling patients or objects), history of chronic diseases, smoking habits, regular physical activity, and over exertional back trauma (resulting from lifting a heavy object or twisting). LBP-related neurological symptoms, medical consultations and medications were inquired about. The body mass index (BMI) was calculated based on self-reported weight and height. The anonymous questionnaire was distributed to the selected practitioners by paper inside closed envelopes.

The Statistical Package for the Social Sciences software, version 22.0 (IBM, SPSS, Chicago, Illinois, USA) was used for entry and analysis of the collected data. Double entry was used to avoid errors. Descriptive statistics were presented as numbers and percentages for categorical data. Univariate and multivariable logistic regression were used to calculate the odds ratios (ORs) and antecedent 95% confidence intervals (95% CIs) for the independent factors for LBP among HCWs in the study. In the multivariable analysis, age and BMI were handled as continuous variables, while other categorical variables were dichotomized. All variables were included in the multivariable analysis.

## Results

### Response rate

Eight hundred questionnaires were distributed. The present study included a total of 740 HCWs who completed the questionnaire, giving a response rate of 92.5%.

### Prevalence of low back pain

The prevalence of LBP in the past 12 months among HCWs is presented in Table 1. The overall prevalence amounted to 73.9% (95% CI: 70.7–77.0). The prevalence of LBP associated with neurological symptoms in the limbs reached 50.0% (95%CI: 46.4–53.6), while the prevalence of LBP necessitating medications and or physiotherapy was 40.5% (95%CI:37.0–44.1). On the other hand, the prevalence of LBP requiring medical consultation in neurosurgical or orthopaedic clinics was only 20% (95%CI: 17.2–23.0). Table 2 shows the overall prevalence of LBP among different HCWs. The highest prevalence was among dentists (88.9, 95%CI: 77.6–98.7) followed by paramedics (74.5, 95%CI: 66.9–81.1), physicians (73.2, 95%CI: 68.6–77.8) and nurses (72.9, 95%CI: 66.6–78.5).

**Table 1:** Prevalence and characteristics of low back pain in the past 12 months among study health care workers (*n* = 740)

Prevalence^a^	No	Prevalence% (95% CI)
Overall prevalence of low back pain	547	73.9 (70.7–77.0)
Prevalence of low back pain with neurological symptoms in limbs	370	50.0 (46.4–53.6)
Prevalence of low back pain necessitating medication and/or physiotherapy	300	40.5 (37.0–44.1)
Prevalence of low back pain requiring medical consultation	148	20.0 (17.2–23.0)

^a^Data were not mutually exclusive

**Table 2:** Univariate analysis of possible risk factors for low back pain (LBP) in the past 12 months among study (*n* = 740) health care workers (HCWs)

Characteristics	Total number of HCWs	LBP in the past 12 months		OR(95%CI)
		No	%	
Level of healthcare				
Primary	176	114	64.8	Ref
Secondary and tertiary	564	433	76.7	**1.80 (1.25–2.59)**
Sex			7	
Male	403	293	2.7	Ref
Female	337	254	75.4	1.15 (0.83–1.60)
Age (years)				
20-	232	158	68.1	Ref
30-	319	255	79.9	**1.87 (1.26–2.75)**
40-	131	93	71.0	1.15 (0.72–1.83
> 50	58	41	70.7	1.13 (0.60–2.12)
Job title				
Physician	353	259	73.2	Ref
Nurse	214	156	72.9	0.99 (0.67–1.45)
Paramedic	145	108	74.5	1.07 (0.69–1.66)
Dentist	27	25	88.9	2.93 (0.86–9.97)
Experience in years				
Less than 5	100	69	69.0	Ref
5–10	283	205	72.4	1.18 (0.72–1.94)
More than 10	357	273	76.5	1.46 (0.90–2.38)
Work conditions				
Long standing	126	86	68.3	Ref
Long sitting	290	225	77.6	**1.61 (1.01–2.56)**
Both	324	236	72.8	1.25 (0.80–1.95)
Body mass index				
Normal weight	282	201	71.3	Ref
Overweight	321	235	73.2	1.10 (0.77–1.57)
Obese	137	111	81.0	**1.72 (1.04–2.83)**
Health status				
No chronic disease	586	428	73.0%	Ref
One or more chronic diseases	154	119	77.3	1.26 (0.83–1.91)
Regular physical activity				
No	227	157	69.2	Ref
Yes	513	390	76.0	**0.70 (0.5–0.99)**
Back Trauma^a^				
No	637	448	70.3	Ref
Yes	103	99	96.1	**10.44 (3.79-28.78)**
Smoking status				
No	656	484	73.8	Ref
Yes	84	63	75.0	1.07 (0.63–1.80)

Bold aOR (95% CI) are statistically significant

*OR* odds ratio for studied factors, *(95% CI)* 95% confidence interval

^a^Overexertional trauma, fall, or lifting heavy objects

### Factors associated with low back pain

Tables 2 and 3 present the univariate and multivariable logistic analyses for the potential personal and work-related factors of LBP in the past 12 months. The univariate analysis showed that the probability of having LBP was significantly higher among HCWs in secondary and tertiary health care hospitals (OR = 1.80, 95% CI:1.25–2.59), HCWs in the age group 30–40 years (OR = 1.87, 95% CI:1.26–2.75), long standing working conditions (OR = 1.61,95% CI:1.01–2.56), being obese (OR = 1.72, 95% CI:1.04–2.83) and positive history of back trauma in the form of over exertional back trauma, falling or lifting heavy objects (OR = 10.44, 95% CI:3.79–28.78). On the other hand, practising regular physical activity was found as a protective factor (OR = 0.70, 95% CI:0.50–0.99). Sex, job title, years of experience, presence of chronic disease and smoking were non-significant factors in having LBP in the past 12 months (Table 2).

**Table 3:** Multivariable logistic regression of risk factors for having back pain in the past 12 months among study health care workers (*n* = 740)

Risk factors	aOR (95% CI)
Secondary and tertiary care vs. primary	**1.32 (1.01–1.76)**
Female vs. male	1.07 (0.72–1.58)
Age in years	1.00 (0.98–1.03)
BMI (kg/m^2^)	**1.10 (1.01–3.65)**
Dentist vs. others	1.10 (0.96–1.26
Long standing vs. others	1.12 (0.92–1.36)
Non-smoker vs. smoker	0.83 (0.46–1.48)
Back trauma^a^ (yes vs. no)	**11.52 (4.14–32.08)**
Physical activity (yes vs. no)	**0.61 (0.42–0.89)**
Chronic disease (yes vs. no)	1.02 (0.89–1.17)

^a^Overexertional trauma, fall, or lifting heavy objects*. aOR* adjusted odds ratio for studied factors, *(95% CI)* 95% confidence interval. Bold aOR (95% CI) are statistically significant

The multivariable logistic regression model confirms the risky effect of working in secondary and tertiary hospitals (aOR = 1.32, 95% CI:1.01–1.76), increased BMI (aOR = 1.10, 95% CI:1.01–3.65), and positive history of back trauma (aOR = 11.52, 95% CI:4.14–32.08) for having LBP in the past 12 months. On the other hand, practising regular physical exercise was a significant protective factor (aOR = 0.61, 95% CI: 0.42–0.89) against the development of LBP (Table 3).

## Discussion

The present study reported an overall 1-year prevalence of LBP among HCWs in southwestern Saudi Arabia of 73.9% (95% CI: 70.7–77.0).

Studies suggest that 1-year prevalence may provide a more precise figure of prevalence, as the recall bias of studied persons is reduced [17]. Similar prevalence figures differ from country to country. In a study among nurses in Taiwan, a prevalence of 72% was found [6]. In Turkey, a figure of 53% of LBP was found among health care workers [18]. Another study in Turkey hospitals reported a prevalence of 77.1% among nurses, 63.3% among physicians and 72.7% among physical therapists [19]. In a study in Riyadh, 65% of nurses reported LBP in the past 12 months [20]. These figures give insight about the global and local magnitude of the problem of LBP among HCWs.

Numerous studies have reported many personal and work-related exposures in the occurrence of LBP among HCWs [5, 6, 9, 10, 12].Due to the disagreements between the outcomes of these studies, it is difficult to find a definite assumption in this regard. Additionally, the variability in LBP magnitude between regions and countries may be explained partly by the difference in the personal criteria as well as the difference in working conditions [21]. The current study offers some evidence concerning the personal and work-related factors associated with LBP among HCWs in southwestern Saudi Arabia.

The present study found a positive association between level of health care (secondary and tertiary vs. primary) and LBP in the past 12 months among HCWs. There are many factors that may be related to the individual’s risk of developing LBP in secondary and tertiary care compared to primary level of care. These factors include stressful working conditions and high workload [6].

The result of the present study confirms the higher risk of LBP among HCWs with a positive history of back trauma in the form of overexertional trauma, falling or lifting heavy objects. Overexertional back trauma is more common among HCWs with long working hours, and patient transfers [22] and is found to be associated with a higher risk of LBP [23].

The current study confirmed the positive association of increased BMI with a higher risk of developing LBP among HCWs. This result is in agreement with the finding of previous studies that reported a higher risk of LBP among obese and overweight HCWs [24, 25].

A recently published meta-analysis study reported a significant relation between BMI and LBP [26]. Overweight and obese people had a higher one-year prevalence of LBP as well as seeking care for LBP compared to those with normal BMI. The majority of the included studies were cross-sectional. Therefore, the association between obesity and LBP could be bidirectional. Obesity and LBP could also be comorbid situations that have common risk factors.

Several possible explanations can clarify this association. First, obesity can exaggerate the mechanical burden on the spine by causing a higher compressive force on the lumbar spine structures during various movements. Obese people may also be more prone to accidents [27]. Second, obesity may trigger LBP through chronic inflammation. Obesity is associated with elevated cytokines and acute-phase reactants and with initiation of proinflammatory pathways [28], which may result in pain [29].The meta-analysis study suggested that obesity is a possibly modifiable risk factor for LBP [26].

The present study revealed a significant protective effect of practising regular exercise on developing LBP in the past 12 months. Similar results have been observed in previous studies [6, 30]. Lack of regular physical exercise results in weak or no back support and incorrect body mechanics [30].

A recently published meta-analysis study suggested that a moderate to high level of physical activity during leisure time protects against frequent or chronic LBP by 11–16% [31]. The explanations lie behind the protective effect of physical exercise against chronic LBP are unclear. Physical exercise interventions for LBP may work by improving posture and muscle activation. However, there is a absence of evidence connecting the effects of exercise in LBP to changes in the musculoskeletal system [32]. There is now solid evidence that LBP is best realised from a biopsychosocial perspective, as it can entail aggregations of different psychological, social, lifestyle and physical factors [33].

The present study also revealed significant factors associated with LBP among HCWs only by univariate analysis, including the age group 20to30years old and longstanding working conditions. This age group represents the most economically active period of working life [34]. A similar age pattern was observed by other studies in different occupational groups [35, 36]. A positive association between long standing working conditions and LBP in the past 12 months was also observed in Taiwan [6], Egypt [37] and Turkey [30]. Long standing could result in a series of musculoskeletal effects, including muscle ischaemia, pain and degeneration of spinal discs [38].However, the lack of evidence for these two variables by multivariate analysis raises many concerns with regard to the reliability of these factors. In the multivariable logistic regression, the factors are mutually adjusted and the alpha level is retained at 0.05. Hence, the results for multivariable analysis are probably more valid and reliable than variables generated from the univariate analysis only.

Study limitations are mostly related to the fact that this study is a cross- sectional exploratory study with an inherent risk of coincidence findings. Another limitation is the fact that the study was done in only one region of southwestern Saudi Arabia. A larger sample size including the other two regions, namely, Jazan and Bisha, may be required to attain more comprehensive results.

## Conclusions

HCWs in southwestern Saudi Arabia are exposed to a highly prevalent LBP health problem. This problem may negatively impact their health and economy. The possible risk factors of LBP are working in higher levels of health care, overexertional back trauma and lack of regular physical exercise.

To minimize the burden of this problem, the physical load should be effectively decreased by proper hospital management and recruiting more staff. Occupational health and safety programmes in hospitals should be implemented to build ergonomically safe working conditions. Encouraging HCWs to practice regular physical exercise will help in decreasing BMI and consequently minimizing LBP incidents.

## Additional file

Additional file 1: Used questionnaire. (PDF 598 kb)

## Abbreviations

aOR: Adjusted odds ratio
BMI: Body mass index
CI: Confidence interval
HCWs: Health care workers
LBP: Low back pain
ORO: Odds ratio
WHO: World Health Organization

## References

[1] Parreira P, Maher C, Steffens D, Hancock M, Ferreira M. Risk factors for low back pain and sciatica: an umbrella review. Spine J. 2018. 10.1016/j.spinee.2018.05.018 29792997

[2] Al-Arfaj AS, Al-Saleh SS, Alballa SR, Al-Dalaan AN, Bahabri SA, Al-Sekeit MA, Mousa MA (2003). How common is back pain in Al-Qaseem region. Saudi medical journal.

[3] Manzini F, Cesana G, Manzini C, Riva MA (2015). A pioneering patient lift: prevention of low Back pain in health care workers in the 18th century. Spine.

[4] Gouveia N, Rodrigues A, Ramiro S, Eusébio M, Machado PM, Canhão H, Branco JC (2017). The use of analgesic and other pain-relief drugs to manage chronic low Back pain: results from a National Survey. Pain Practice.

[5] Leijon O, Wiktorin C, Härenstam A, Karlqvist L, Group MR (2002). Validity of a self-administered questionnaire for assessing physical work loads in a general population. J Occup Environ Med.

[6] Shieh S-H, Sung F-C, Su C-H, Tsai Y, Hsieh VC-R (2016). Increased low back pain risk in nurses with high workload for patient care: a questionnaire survey. Taiwanese Journal of Obstetrics and Gynecology.

[7] Al Dajah S, Al Daghdi A. Prevalence and risk factors of low back pain among nurses in Sudayr region. European Scientific Journal, ESJ. 2013;9(33).

[8] Holtermann A, Clausen T, Aust B, Mortensen OS, Andersen LL (2013). Risk for low back pain from different frequencies, load mass and trunk postures of lifting and carrying among female healthcare workers. Int Arch Occup Environ Health.

[9] Awaji M (2016). Epidemiology of low Back pain in Saudi Arabia. J Adv Med Pharm Sci.

[10] Johnson OE, Edward E (2016). Prevalence and risk factors of low Back pain among Workers in a Health Facility in south–South Nigeria.

[11] Dario AB, Ferreira ML, Refshauge KM, Lima TS, Ordoñana JR, Ferreira PH (2015). The relationship between obesity, low back pain, and lumbar disc degeneration when genetics and the environment are considered: a systematic review of twin studies. Spine J.

[12] Behisi MA, Al-Otaibi ST, Beach J (2013). Back pain among health care workers in a Saudi Aramco facility: prevalence and associated factors. Arch Environ Occup Health.

[13] Homaid MB, Abdelmoety D, Alshareef W, Alghamdi A, Alhozali F, Alfahmi N, Hafiz W, Alzahrani A, Elmorsy S (2016). Prevalence and risk factors of low back pain among operation room staff at a tertiary care center, Makkah, Saudi Arabia: a cross-sectional study. Annals of occupational and environmental medicine.

[14] Lwanga SK, Lemeshow S, Organization WH (1991). Sample size determination in health studies: a practical manual.

[15] Lin P-H, Tsai Y-A, Chen W-C, Huang S-F (2012). Prevalence, characteristics, and work-related risk factors of low back pain among hospital nurses in Taiwan: a cross-sectional survey. Int J Occup Med Environ Health.

[16] Koes B, Van Tulder M, Thomas S (2006). Diagnosis and treatment of low back pain. Bmj.

[17] Glover W (2002). Work-related strain injuries in physiotherapists: prevalence and prevention of musculoskeletal disorders. Physiotherapy.

[18] Şimşek Ş, Yağcı N, Şenol H (2017). Prevalence and risk factors of low Back pain among health-care Workers in Denizli. Ağrı-The Journal of The Turkish Society of Algology.

[19] Karahan A, Kav S, Abbasoglu A, Dogan N (2009). Low back pain: prevalence and associated risk factors among hospital staff. J Adv Nurs.

[20] Abbas MA, ZAID LZA, FIALA LA, NA ALHAMDAN. Prevalence and risk factors of low back pain among nurses in four tertiary care hospitals at king Fahad Medical City, Riyadh. **KSA** *The Medical Journal of Cairo University*. 2010;78(2).

[21] Anderson SP, Oakman J (2016). Allied health professionals and work-related musculoskeletal disorders: a systematic review. Safety and health at work.

[22] Engkvist I-L, Hjelm EW, Hagberg M, Menckel E, Ekenvall L: Risk indicators for reported over-exertion back injuries among female nursing personnel. In: LWW; 2000. 10.1097/00001648-200009000-00006 10955403

[23] Hoy D, Bain C, Williams G, March L, Brooks P, Blyth F, Woolf A, Vos T, Buchbinder R (2012). A systematic review of the global prevalence of low back pain. Arthritis & Rheumatology.

[24] Jensen JN, Holtermann A, Clausen T, Mortensen OS, Carneiro IG, Andersen LL (2012). The greatest risk for low-back pain among newly educated female health care workers; body weight or physical work load?. BMC Musculoskelet Disord.

[25] Mirtz TA, Greene L (2005). Is obesity a risk factor for low back pain? An example of using the evidence to answer a clinical question. Chiropractic & osteopathy.

[26] Shiri R, Karppinen J, Leino-Arjas P, Solovieva S, Viikari-Juntura E (2009). The association between obesity and low back pain: a meta-analysis. Am J Epidemiol.

[27] Hu H, Chou Y-J, Chou P, Chen L, Huang N (2009). Association between obesity and injury among Taiwanese adults. Int J Obes.

[28] Tilg H, Moschen AR (2006). Adipocytokines: mediators linking adipose tissue, inflammation and immunity. Nat Rev Immunol.

[29] Shiri R, Solovieva S, Husgafvel-Pursiainen K, Taimela S, Saarikoski LA, Huupponen R, Viikari J, Raitakari OT, Viikari-Juntura E (2008). The association between obesity and the prevalence of low back pain in young adults: the cardiovascular risk in young Finns study. Am J Epidemiol.

[30] Terzi R, Altın F (2015). The prevalence of low back pain in hospital staff and its relationship with chronic fatigue syndrome and occupational factors. Agri: Agri (Algoloji) Dernegi'nin Yayin organidir= The journal of the Turkish Society of Algology.

[31] Shiri R, Falah-Hassani K: Does leisure time physical activity protect against low back pain? Systematic review and meta-analysis of 36 prospective cohort studies. Br J Sports Med 2017:bjsports-2016-097352. 10.1136/bjsports-2016-097352 28615218

[32] Halliday MH, Pappas E, Hancock MJ, Clare HA, Pinto RZ, Robertson G, Ferreira PH (2016). A randomized controlled trial comparing the McKenzie method to motor control exercises in people with chronic low back pain and a directional preference. J Orthop Sports Phys Ther.

[33] Kamper SJ, Apeldoorn A, Chiarotto A, Smeets R, Ostelo R, Guzman J, Van Tulder M (2015). Multidisciplinary biopsychosocial rehabilitation for chronic low back pain: Cochrane systematic review and meta-analysis. Bmj.

[34] Maniadakis N, Gray A (2000). The economic burden of back pain in the UK. Pain.

[35] Walker BF (2000). The prevalence of low back pain: a systematic review of the literature from 1966 to 1998. Clinical Spine Surgery.

[36] Mehrdad R, Shams-Hosseini NS, Aghdaei S, Yousefian M (2016). Prevalence of low back pain in health care workers and comparison with other occupational categories in Iran: a systematic review. Iranian journal of medical sciences.

[37] Hegazy A, Awadalla N, Shenouda N (2009). Prevalence of musculoskeletal complaints among dentists in some Egyptian cities. Egyptian Journal of Occupational Medicine.

[38] Valachi B, Valachi K (2003). Mechanisms leading to musculoskeletal disorders in dentistry. J Am Dent Assoc.

