# Supplementary material for: Prevalence and factors associated with low back pain among health care workers in southwestern Saudi Arabia
Source: BMC Musculoskelet Disord. 2019 Feb 8;20:56. doi: 10.1186/s12891-019-2431-5 (PMC6368758; doi:10.1186/s12891-019-2431-5)
Supplement: Supplementary file 1 — Used questionnaire. (PDF 598 kb) [file 12891_2019_2431_MOESM1_ESM.pdf]

Abha Summer School
for Health Research
مدرسة أبها الصيفية للأبحاث الصحية

Questionnaire Back Pain

Dear healthcare worker, The following questionnaire aims To estimate the prevalence of back pain among health care workers, southern region, Saudi Arabia. We would be very grateful if you could help us by completing this questionnaire. Filling this questionnaire means agreement to participate in the study.

1. Name of the Hospital/PHCC.......
2. Gender   Male/Female
3. Age ....
4. Nationality   Saudi /non Saudi
5. Length ....cm   Weight ....Kg.   Dominant hand (Right / Left)
6. Profession: Physician: general practitioner /registrar / resident / consultant
   Nurse: practical / specialist
   Lab Technician / specialist
   Radiologist   Pharmacologist   Psychologist
   social worker   Dentist   Other..................
7. Department: Medical / specialty................
   Surgical / specialty ....
   ER   OPD   ICU   IMCU   Home visit
   Pharmacy
   Dental center   Lab   Other: ....
8. Years of Work ......
9. Work status: Long standing /Sitting /Both)
10. Smoking Yes / No
11. Do you have bake pain? yes/ no   if Yes How long? ....
   did you Need a Drug to relieve a pain? Yes/No,   if yes whit type of drug did use?.................
12. If you have back pain which part of your back: cervical(neck) thoracic(middle to upper back) Lumper(lower back)
13. If have back pain do you Need to take a break time: Yes (How long) / No
14. Did you do exercises to relieve pain? Yes/ no   if yes which type of exercises do you do?............
15. Did you visit a Neurosurgical Clinic due to Back pain Yes / No
16. Do have any Chronic Disease:(DM. HTN. ASTHMA. RENAL.) Other.................
17. History of back trauma: Yes (since ....) / No
18. Other neurological symptoms on your limb   Pain /Numbness/ Weakness
19. Are you an Athlet Yes / NO
